# A Call to Action to Public Health Institutions and Teaching to Incorporate Mass Incarceration as a Sociostructural Determinant of Health

**DOI:** 10.1177/00333549221120243

**Published:** 2022-09-05

**Authors:** Erin J. McCauley, Katherine LeMasters, Michael F. Behne, Lauren Brinkley-Rubinstein

**Affiliations:** 1 Department of Social and Behavioral Sciences, University of California San Francisco, San Francisco, CA, USA; 2 Philip R. Lee Institute for Health Policy Studies, University of California San Francisco, San Francisco, CA, USA; 3 Department of Epidemiology, Gillings School of Global Public Health, University of North Carolina at Chapel Hill, Chapel Hill, NC, USA; 4 Re-Envisioning Health & Justice Lab, Department of Social Medicine, University of North Carolina at Chapel Hill, Chapel Hill, NC, USA; 5 Department of Social Medicine, University of North Carolina at Chapel Hill, Chapel Hill, NC, USA; 6 Center for Health Equity Research, University of North Carolina at Chapel Hill, Chapel Hill, NC, USA

**Keywords:** mass incarceration, public health, curricula

Mass incarceration refers to the system of social and racial control in the United States that arrests, convicts, incarcerates, and supervises racial and ethnic minority populations through probation and parole. Mass incarceration is referred to as “mass” because the current size of this system in the United States is historically and internationally unparalleled. Mass incarceration affects those who are incarcerated and under community supervision, as well as the families and communities where it is concentrated.^1^ Mass incarceration is a pervasive cause of health inequities in the United States.^2^ Yet, it has long been absent from both public health institutional priorities and core graduate training in public health. Mass incarceration is absent from the required curricula in general public health programs and even specialty programs focused on health equity and sociostructural determinants of health. Given this lack of prioritization and formal education and training, public health scholars may overlook the critical role it plays in individual and community health and the key it holds to achieving health equity. Increased broad awareness of the harms of mass incarceration is needed to disrupt its connection to health inequities and to abolish this system of social and racial control.

In the United States in 2020, the criminal legal system directly affected almost 7 million people daily, with 2.3 million people incarcerated in state and federal prisons, local jails, and Immigrations and Customs Enforcement detention facilities, and 4.4 million under community supervision.^3^ Criminal legal involvement is not random: Black, American Indian/Alaska Native, Native Hawaiian and Other Pacific Islander, and Latinx people with a high school education or lower have a higher likelihood of criminal legal involvement than their non-Hispanic White and more highly educated counterparts.^3^ In addition, while men make up the majority of those incarcerated, women are the fastest-growing segment of the prison population, and non-White women are disproportionately represented.^3^ Familial incarceration is also common and unequally distributed; 1 in 3 young people will have a parent who is incarcerated, and 45% of people in the United States will have an immediate family member who is incarcerated.^4^

People experiencing incarceration at any point in life have disproportionately poor physical health (eg, increased HIV incidence) and mental health (eg, trauma) before, during, and after incarceration.^2^ This disproportion was especially evident during the COVID-19 pandemic. In prisons, between April 2021 and April 2022, the rate of confirmed COVID-19 cases was 5 times greater and the mortality rate was 3 times greater than in the general population.^5^ The COVID-19 pandemic has also highlighted how the criminal legal system and population health are intricately linked. Throughout the pandemic, COVID-19 spread in carceral facilities contributed substantially to community spread and vice versa,^6,7^ underscoring the importance of mass incarceration to community health and health equity. Beyond COVID-19, incarceration also harms the health of nonincarcerated family members through increased stress and trauma, widespread stigma, and financial deprivation.^8^ Mass incarceration erodes community mental health and alters the social ecology of neighborhoods by eroding social capital and disrupting social and family networks.^9^ The residents of these neighborhoods also experience poor physical health and have a high prevalence of asthma and sexually transmitted infections.^10,11^ Given the widespread and disproportionate reach of the criminal legal system and its many direct and indirect effects on health, this system contributes to extreme health inequities.

## Recommendation 1: Increased Focus on the Criminal Legal System by Public Health Institutions, Departments, and Funders

There has been a historical lack of focus on the criminal legal system in public health institutions and departments and among funders. In 2021, the American Public Health Association (APHA) called for decarceration—the intentional reduction of the incarcerated population in the United States through both releases and decreases in admissions—and divestment from carceral systems as a public health solution. APHA’s statement explained that decarceration would improve the health of those currently and formerly incarcerated and their families and communities through (1) divesting from carceral systems and investing in other sociostructural determinants of health (eg, housing, employment), (2) committing to noncarceral measures for accountability and safety, (3) restoring voting rights to formerly and currently incarcerated people, and (4) funding research to evaluate policy determinants of exposure to the carceral system and proposed alternatives.^12^ However, to achieve decarceration and focus on mass incarceration as a public health problem, additional steps are needed beyond a single statement, and public health as a discipline must consider mass incarceration to be a public health priority and decarceration to be a public health solution.

Public health institutions, including APHA, must make structural changes to reflect their renewed focus on and prioritization of the criminal legal system. The criminal legal system is absent from the core topics and issues around which the APHA is organized. APHA’s core topics and issues list includes other domains reflecting sociostructural determinants of health, such as high school graduation, immigration, and climate change. Under the topics and issue domain of the sociostructural determinants of health, education, housing, and income inequality are highlighted, but the criminal legal system is not mentioned. Furthermore, the criminal legal system is missing from its member sections (the organization’s primary professional units) and from its caucuses, which focus explicitly on social justice issues.

State and county departments of health employ many public health professionals and have a duty to protect the public’s health, yet they fail to prioritize the criminal legal system. For example, collaborations between departments of health and departments of corrections are critical to reducing the spread of disease in facilities and improving community health, but these partnerships are rare and limited in scope.^13^ Furthermore, when these partnerships do exist, they often focus on disease outbreaks rather than decarceration, which is necessary for sustainably improving health on a population level.^14^ Departments of health must increase their collaborations with the criminal legal system and advocate for decarceration to promote population health.

The absence of the criminal legal system is apparent in funding systems as well. The National Institutes of Health (NIH) does not consider criminal legal–involved populations as a health disparities population, defined as a population experiencing higher rates of disease and death than the general population. Yet, this population does experience high rates of disease (eg, psychiatric illness, infectious disease) and premature death. Also, criminal legal involvement disproportionately affects Black, low-income, and medically underserved rural communities, all of whom are considered health disparities populations and are prioritized for funding from the National Institute on Minority Health and Health Disparities. Furthermore, the only NIH branch that prioritizes criminal legal involvement is the National Institute on Drug Abuse. While untreated substance use disorders are highly prevalent in the criminal legal–involved population, it is not this population’s only health issue. The lack of attention to the criminal legal system has resulted in less than 0.1% of NIH funding being focused on criminal legal research.^15^ NIH must holistically incorporate criminal legal–involved populations into its priorities.

## Recommendation 2: Revise Public Health Curriculum to Incorporate the Criminal Legal System

The lack of prioritization of the criminal legal system is evident in public health teaching as well. Mass incarceration and the criminal legal system are currently taught as a niche interest of relevance for individual researchers and subfields rather than as a primary topic that shapes public health. For example, public health curricula often do not include the criminal legal system as a sociostructural determinant of health in introductory public health courses, and the criminal legal system is missing from most models of the sociostructural determinants of health.^16^ Courses on mass incarceration and public health are offered at some schools and programs of public health but are not required, further isolating this topic and population from mainstream learning. Public health curricula must be revised to instruct all students on the prominent role of the criminal legal system in health equity and its function as a sociostructural determinant of health, thereby centering the criminal legal system in public health.

Furthermore, public health programs do not include training in understanding the criminal legal system structure and accessing and managing criminal legal system data, which is imperative in fostering research on the health consequences of the criminal legal system. The criminal legal system is labyrinthine and deliberately confusing. The system (1) exists at the federal, state, and county levels; (2) includes private and public facilities; (3) has juvenile, adult, and immigration divisions; and (4) separates tribal jails to distinct jurisdictions. This complex structure has implications for collaborations with health departments and health care entities,^13^ access to services (eg, Medicaid) during and after incarceration,^17^ and funding.^18^ This structure also has implications for what data are collected from criminal legal systems and how they are made available—dictating if and how researchers can access the data. While schools and programs of public health often offer courses on working with data from health care and other databases, they lack courses on accessing and using data from criminal legal systems, further making these data difficult to use. Knowing where and how to access these data is necessary for using this information and must be taught to public health students. By not doing so, we are unintentionally creating a public health workforce that is unprepared for this work. Including mass incarceration as a core component of public health curriculum will advance both public health and the public health discipline by equipping future scholars and practitioners with the knowledge and skills needed to study and address its role in the development and persistence of health inequity.

## Recommendation 3: Prioritize Employment for Formerly Incarcerated People

A critical component for prioritizing the criminal legal system in public health institutions is emphasizing the voice of those with lived experience in the system through employment. However, formerly incarcerated people and those under community supervision face profound employment barriers in public health institutions. These barriers amplify why criminal legal issues are viewed as niche interests in public health: those with lived experience are barred from entering. Many states prohibit formerly incarcerated people from being drug counselors or prohibit people on probation or parole from talking to others with criminal records.^19-21^ These prohibitions are in place despite ample evidence that peer support programs are proven to lead to improved outcomes among people exiting incarceration.^22^

Many universities have policies that bar the enrollment or employment of formerly incarcerated people or introduce onerous barriers.^23^ Recent policy changes to reduce barriers to employment for the formerly incarcerated, such as Ban the Box efforts, may delay background checks and thereby reduce some barriers to employment.^24^ However, universities and other public health institutions must go beyond reducing barriers to employment and should actively seek to employ formerly incarcerated people to leverage their lived experiences and intimate knowledge of the criminal legal system, as well as support their re-entry transition. Universities have previously prioritized employment groups. For example, in 2011, when the University of California San Francisco expanded its campus, the university made a commitment to hire at least 20% of workers locally to leverage its power to improve the local economy, expand training opportunities in the community, and increase local capacity.^25^ Similar efforts should be undertaken to prioritize the employment of formerly incarcerated people in universities and public health organizations. In addition, employers must provide flexibility to make sustained employment possible, because parole and probation require regular appointments at parole and probation offices during the workday. Prioritized employment would leverage the power of these institutions to center the lived experience of incarceration, provide opportunities for training and advancement to formerly incarcerated people, and promote health equity and social equality through increasing access to flexible, dependable, and well-paid employment with benefits to a group of people who historically have been socially and economically marginalized.

## Public Health Implications

Mass incarceration will continue to create and exacerbate health inequities until our institutions prioritize mass incarceration as a sociostructural determinant of health and meet APHA’s call to decarcerate. We must (1) revise the priorities of our public health institutions, departments of health, and funders to reflect this renewed understanding of the centrality of the criminal legal system for public health; (2) revise public health curricula to include the criminal legal system as a primary sociostructural determinant of health in introductory courses and include required training on the criminal legal system and criminal legal data; and (3) reduce barriers to employment in the field of public health for formerly incarcerated people. Mass incarceration is a sociostructural determinant of health that must be holistically incorporated into public health institutions and teaching. Efforts to teach, research, and promote health equity must focus on the role that mass incarceration plays and the need to dismantle and reimagine our criminal legal system.
